# Smartphone-Based Interpretable Machine Learning for Classifying Single-Leg Squat Performance Using Trunk, Pelvic, and Knee Kinematics: Cross-Sectional Study

**DOI:** 10.2196/85126

**Published:** 2026-03-12

**Authors:** Sihyun Kim, Kyuenam Park

**Affiliations:** 1 Department of Physical Therapy, Sangji University, Wonju, Republic of Korea; 2 Digital Healthcare and Sports Data Science Lab, Department of Physical Education, Yonsei University, 50 Yonsei-ro, Seodaemun-gu, Seoul, 03722, Republic of Korea, 82 02-2123-6192

**Keywords:** explainable artificial intelligence, XAI, movement quality assessment, single-leg squat performance, smartphone-based assessment, supervised machine learning

## Abstract

**Background:**

Single-leg squat (SLS) performance is widely used to screen functional movement quality, but practical assessment often relies on expert visual grading or laboratory-based motion capture. In addition, conventional SLS criteria mainly focus on isolated joint deviations and may overlook coordination-related, multisegment movement patterns that characterize impaired performance.

**Objective:**

This study aimed to examine the feasibility of an interpretable machine learning framework for classifying SLS performance into 3 levels (good, moderate, and poor) from single-smartphone, frontal-view videos based on trunk, pelvic, and knee kinematics, and to evaluate coordination-informed features and model explainability using Shapley additive explanations (SHAP) and local interpretable model-agnostic explanations (LIME).

**Methods:**

A dataset of frontal-view SLS videos was labeled by physiotherapists into 3 functional categories (good, moderate, and poor). Videos were processed using 2D pose estimation, and models were trained on 17 engineered kinematic features derived from trunk, pelvic, and knee angles. Following the feature selection, 7 classifiers were trained and evaluated using the 8 selected features with stratified 5-fold cross-validation and a held-out test set. SHAP and LIME were applied for global and local interpretability.

**Results:**

On the held-out test set, adaptive boosting classified SLS performance with an accuracy of 0.84, an *F*_1_-score of 0.85, and an area under curve of 0.92. SHAP indicated that the summated angle (trunk + pelvis + knee), coordination-related features (knee × trunk interaction and knee-to-trunk ratio), and knee angle were key contributors to model predictions. LIME provided instance-level explanations that helped interpret individual classifications and decision boundaries.

**Conclusions:**

This study presents an interpretable machine learning framework for classifying SLS performance into 3 levels using frontal-view videos acquired with a single smartphone. By leveraging coordination-informed engineered features and explainable artificial intelligence, the framework enables transparent interpretation of movement performance beyond isolated joint deviations. The proposed workflow uses smartphones for standardized video acquisition, while performance screening is achieved through machine learning. Given its lightweight feature design, this framework has potential for future implementation on modern smartphones and may support rehabilitation planning and injury-prevention strategies in sports and clinical settings.

## Introduction

Knee function is a key factor in mobility, athletic performance, and quality of life in active young adults, and knee-related problems are common in this population [1]. In young physically active populations, functional performance tests provide meaningful evaluations of knee function by indirectly estimating muscle strength, joint stability, flexibility, balance, and proprioception, thereby offering insights that are not available through static radiographic imaging [2,3]. Among these, the single-leg squat (SLS) is widely used to assess dynamic lower extremity and trunk movement quality in both sports and clinical contexts, with movement quality rated on either a 2-point (good vs poor) or a 3-point ordinal scale (good vs moderate vs poor) [4]. SLS performance is typically evaluated via expert visual observation of segmental alignment or using laboratory-based 3D motion capture to assess hip internal rotation, knee valgus, trunk lateral lean, and pelvic drop [5,6]. 3D motion capture can measure movement with high accuracy, but it requires costly equipment and substantial processing time compared to visual assessment [7]. Visual assessment, although less expensive, still necessitates expert analysis of recorded footage, making it time-consuming and susceptible to observer bias and limited reproducibility when classifying movement quality [5].

Recently, machine learning algorithms have been applied to evaluate and classify SLS performance without costly 3D motion capture systems or expert visual assessments, thereby reducing analysis time [7,8]. An inertial measurement unit (IMU)-based support vector machine (SVM) model using 3 sensors achieved 96% accuracy for binary classification (good vs poor) but decreased to 66% for 3-class classification (good vs moderate vs poor) based on hip and knee angles [9]. However, IMU accuracy depends on the number of sensors, and the requirement for multiple sensors limits clinical feasibility [9]. More recent approaches using consumer-grade cameras with pose estimation algorithms have achieved moderate-to-high accuracy in 3-class SLS classification, even with a single camera [7,10]. For example, pose-estimated SLS videos captured with a single camera achieved 53%‐77% agreement with expert assessments across 6 postural orientation errors, with knee valgus errors showing the highest agreement (77%) [7]. Similarly, a smartphone-based markerless motion capture study found that trunk, pelvic, and knee angles, together with the summated angle of these joints, enabled 3-class classification with 76.9% accuracy using a decision tree model [10]. However, these studies primarily extracted features based on expert-defined SLS guidelines, such as trunk lateral leaning and knee valgus, whereas SLS performance involves global, coordinated movement patterns of the trunk and lower limbs. It is necessary for future machine learning classification models to generate features that capture interjoint movement patterns, such as the knee-to-trunk ratio and joint interaction terms during the SLS test.

While recent machine learning approaches have demonstrated promising accuracy in SLS classification, the underlying decision-making mechanisms often remain unclear, which limits their adoption in clinical practice [11]. In rehabilitation and sports medicine, clinicians and other professionals need to understand which specific kinematic factors drive model predictions in order to design targeted interventions and maintain clinical confidence [12]. Explainable artificial intelligence (XAI) methods, such as Shapley additive explanations (SHAP) and local interpretable model-agnostic explanations (LIME), can provide feature-level explanations by quantifying the contribution of each joint movement or interaction to the model’s decision [13]. Applying XAI to SLS performance assessment can both support movement quality classification and identify key movement patterns, bridging algorithmic predictions with clinical feedback and fostering trust and personalized rehabilitation planning.

In this study, we aim to examine the feasibility of an interpretable machine learning framework for classifying SLS performance based on trunk, pelvic, and knee kinematics into 3 levels (good, moderate, and poor) using frontal-view SLS videos recorded with a single smartphone. Using biomechanically informed feature engineering and hybrid feature selection, we further investigate whether coordination-related feature combinations (ratio, interaction, and summated features) involving trunk, pelvic, and knee angles provide clinically meaningful representations of movement quality beyond conventional single-joint angle metrics. In addition, we evaluate model interpretability using SHAP and LIME to support transparent functional grading and facilitate clinical translation in sports and rehabilitation screening.

## Methods

### Participants

**Figure 1.**
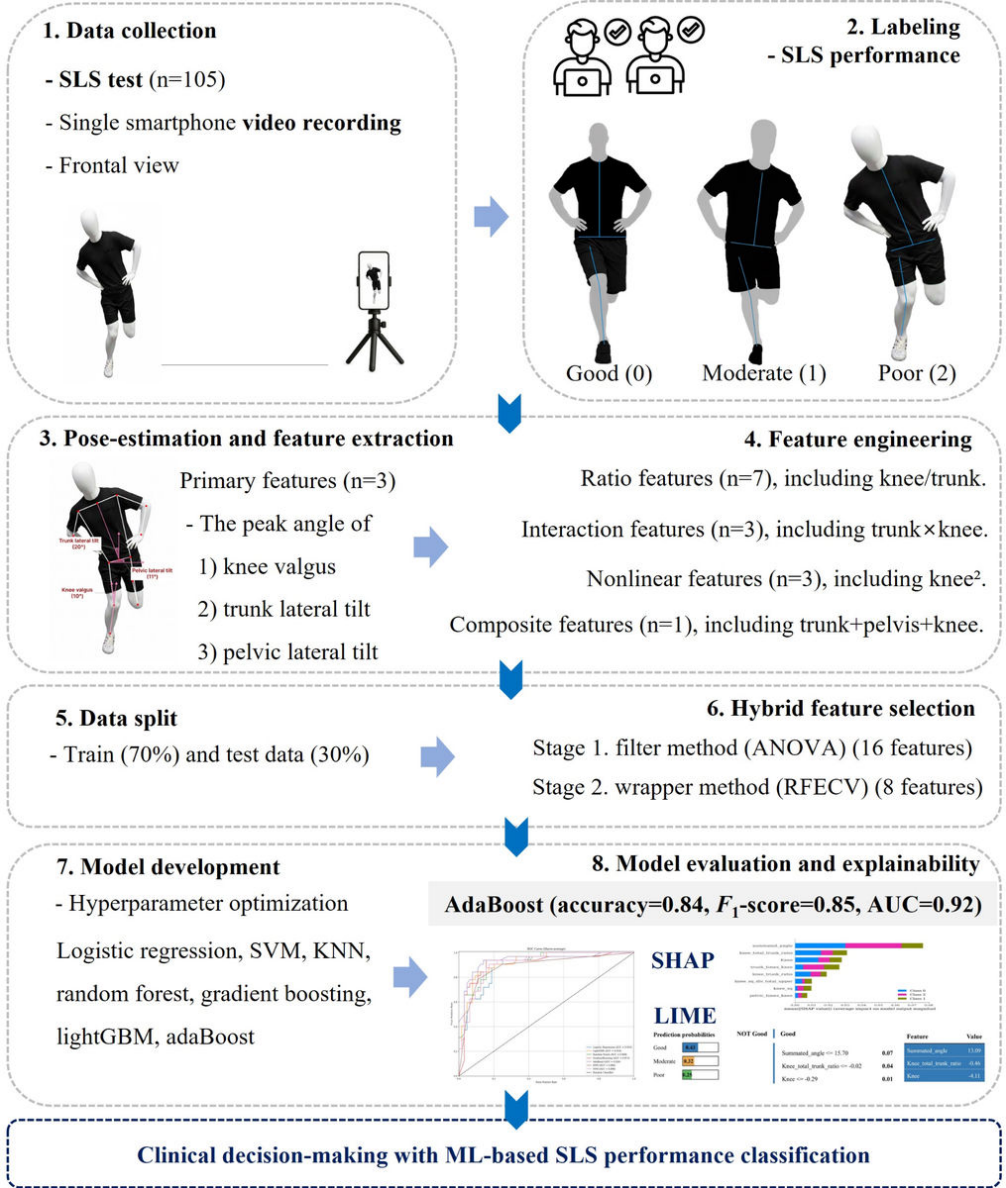
Flowchart of the machine learning model development process for SLS performance classification and interpretable feedback. AdaBoost: adaptive boosting; AUC: area under curve; KNN: *k*-nearest neighbors; LightGBM: light gradient boosting machine; LIME: local interpretable model-agnostic explanations; ML: machine learning; RFECV: recursive feature elimination with cross-validation; SHAP: Shapley additive explanations; SLS: single-leg squat; SVM: support vector machine.

**Figure 2.**
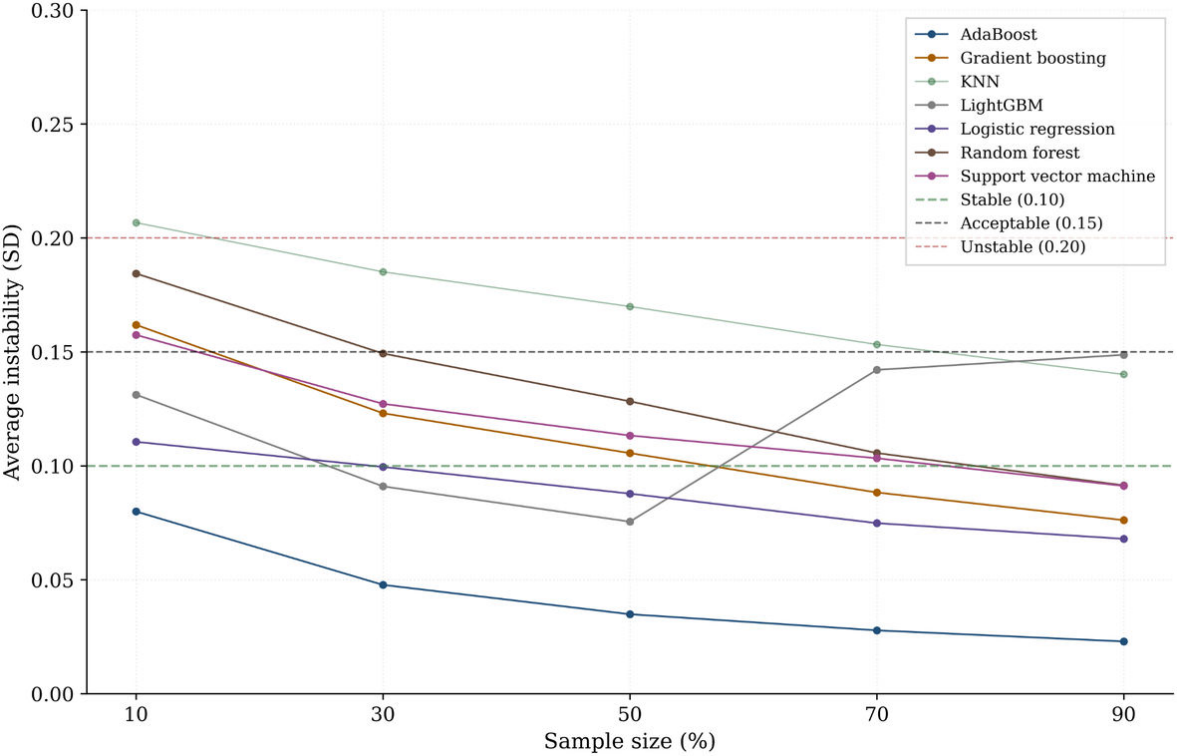
Prediction instability across varying sample sizes for multiple machine learning models evaluated using bootstrap resampling. AdaBoost: adaptive boosting; KNN: *k*-nearest neighbors; LightGBM: light gradient boosting machine.

An overview of the overall study workflow is presented in Figure 1. This study included a total of 105 young active adults (aged 19‐30 y) who were recruited through university campus advertisements, social media, and local community bulletin boards. Participants were eligible for inclusion if they engaged in regular physical activity for at least 60 minutes per week. Exclusion criteria included a history of lower-extremity or low back pain within the previous 3 months, prior major knee injuries or surgeries, neurological disorders, or an inability to perform the SLS test [14]. Sample size adequacy for machine learning model development was evaluated using a prediction instability approach. Prediction instability was assessed across incremental proportions of the dataset (10%, 30%, 50%, 70%, and 90%), where instability was defined as the variability of model performance across resampled subsets [15]. The analysis showed a marked reduction in prediction instability with increasing sample size, with the final sample of 105 SLS videos yielding stable model performance across the evaluated machine learning models. Bootstrap resampling with 100 iterations indicated that using more than 70% of the dataset resulted in acceptable prediction stability, as reflected by low instability values, supporting the adequacy of the sample size for reliable machine learning performance (Figure 2).

### Data Collection

Videos were recorded using an iPhone, mounted on a tripod positioned 3 meters in front of the participant at pelvic height, capturing the entire body at 30 Hz and 1080 p resolution. Only the frontal view was recorded, as clinical evaluation of SLS performance primarily relies on frontal plane deviations including trunk lean, pelvic tilt, and medial knee displacement [7]. Additionally, this single-camera configuration reflects a practical and scalable approach for real-world clinical assessment settings, eliminating the need for multiple camera setups to capture additional views (side or top) [16].

**Figure 3.**
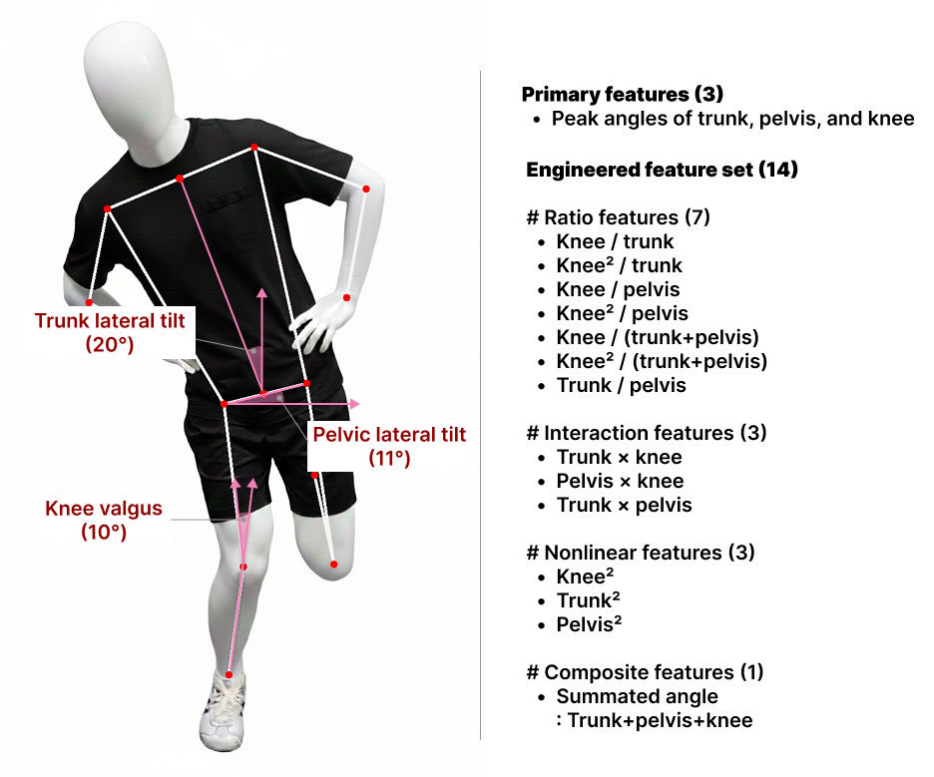
Representative single-leg squat frame and interpretable kinematic features used for model training.

Each participant performed the SLS on their dominant leg (defined as the leg used to kick a ball), wearing standard gym clothing (eg, shorts and t-shirts) to ensure full visibility of lower-limb motion. Participants were given a brief warm-up consisting of 5 minutes of stationary cycling and performed 3 practice trials under the supervision of a physiotherapist to ensure consistent technique. The SLS movement was performed at a comfortable, self-selected pace, avoiding excessively slow speed that might reduce compensatory kinematic deviations such as knee valgus [17]. The standardized SLS test procedure was as follows: (1) Participants stood with their feet shoulder-width apart and toes pointing forward; (2) Participants then placed their hands on the waist and extended the nondominant leg backward, maintaining approximately 90° of knee flexion; (3) From this position, participants descended into a squat as deeply as possible at a self-selected speed, bending the weight-bearing leg without losing balance, maintaining an upright posture, and facing the front camera (Figure 3); and (4) Finally, they fully extended the knee of the weight-bearing leg to return to the starting position [18].

Each participant performed 3 repetitions, with a 1-minute rest between attempts. A repetition was considered invalid if the participant lost balance, allowed the legs to touch, placed the nondominant foot on the ground, or failed to maintain an upright posture facing the camera. In such cases, an additional repetition was performed at the end of the trial. If 2 or more repetitions within a trial were invalid, the entire trial was repeated after a sufficient rest period [17].

### Labeling of Single-Leg Squat Movement Quality

Movement quality was evaluated independently by 2 licensed physiotherapists, each with over 10 years of clinical experience in musculoskeletal rehabilitation. Prior to labeling, the evaluators completed a structured 2-hour training session on SLS assessment criteria, which involved repeatedly reviewing and grading 5 representative SLS video recordings excluded from the main dataset [19]. During this session, a consensus discussion with one of the authors was conducted to standardize the interpretation of movement patterns and rating procedures, thereby ensuring consistent and objective classification.

Following training, the 2 evaluators independently assessed movement quality through visual observation while blinded to participant characteristics and not involved in other experimental procedures. Each participant’s performance was reviewed no more than 2 times using full-length SLS videos played at normal speed on a personal computer, without pausing, rewinding, or slow-motion playback [19]. This approach was informed by previous findings indicating that even when raters are allowed unlimited repeated viewing in both real-time and slow-motion conditions, interrater reliability does not necessarily improve beyond fair to moderate levels for several movement components [20]. During each review, performance was assessed using a multisegmental approach, simultaneously evaluating the trunk, pelvic, and knee segments across 3 consecutive squats [19]. SLS performance was graded into 1 of 3 categories based on observable frontal-plane deviations [21]. Class 0 (good) was defined by no lateral trunk or pelvic tilt, absence of visible knee valgus, and a stable, straight knee trajectory throughout the squat. Class 1 (moderate) included mild lateral trunk or pelvic tilt with slight knee valgus and minor medial-lateral knee movement without notable instability. Class 2 (poor) was characterized by pronounced lateral trunk or pelvic tilt, obvious knee valgus, and clear mediolateral displacement or shaking of the knee.

Each repetition was independently and blindly assessed by 2 evaluators. Agreement scores were accepted when ratings matched. In cases of disagreement, the evaluators re-reviewed the video recording, shared their initial ratings, and discussed the observed movement patterns to reach agreement on a final score [7]. Before consensus, interrater agreement for the 3-level SLS performance grading was assessed using weighted Cohen Kappa, indicating a moderate level of agreement (*κ*=0.60).

The final class label for each participant was defined as the poorest performance observed across the 3 repetitions [7,21]. The poorest SLS repetition was used for machine learning training to represent participant-level movement quality because clinical screening prioritizes detection of the most impaired movement pattern. In addition, when multiple repetitions from a single participant receive different labels, trial-level modeling can introduce ambiguity in participant-level classification. When multiple repetitions received the same label, the first valid repetition was used for pose estimation and feature extraction to ensure a deterministic and reproducible trial-selection rule and to minimize potential learning and fatigue effects across repeated trials [22].

### Pose Estimation and Feature Extraction

For consistency between labeling and analysis, the same poorest-rated trial used for labeling was also selected for 2D pose estimation and feature extraction. Pose estimation of the SLS smartphone video was performed using the MediaPipe Pose library (v0.10.21) (Google), which detects 33 body landmarks from RGB frames in real time. MediaPipe Pose operates without the need for depth sensors, specialized hardware, or manual landmark initialization, making it well-suited for smartphone-based clinical assessments. Previous research has demonstrated that MediaPipe Pose provides highly valid estimates of lower-limb joint angles compared with gold-standard 3D motion capture (Pearson correlation=0.87) and exhibits excellent test-retest reliability (intraclass correlation coefficient=0.93‐0.95), supporting its suitability for clinical biomechanics applications [23].

In accordance with established SLS test guidelines [21], trunk, pelvic, and knee angles were computed frame by frame across the entire SLS movement cycle. For each joint, the peak deviation from neutral alignment was extracted and used to quantify frontal-plane deviations during the SLS test. Knee valgus was computed as 180° minus the internal angle formed by the hip, knee, and ankle landmarks, representing frontal-plane knee alignment. Trunk lateral tilt was defined as the maximum deviation angle between the vertical axis and the line connecting the shoulder and hip midpoints, representing upper body lean. Pelvic lateral tilt was measured as the maximum angle between the line connecting the left and right hip landmarks and the horizontal axis, reflecting pelvic obliquity (Figure 3).

While the primary joint angles align with conventional SLS evaluation criteria [21], they may overlook complex inter-segmental relationships and compensatory movement strategies. To expand the feature representation beyond the primary measures, 3 frontal-plane joint angles (trunk, pelvic, and knee) were used to derive 14 engineered kinematic features. The engineered feature set included ratio features (knee/trunk, knee²/trunk, knee/pelvis, knee²/pelvis, knee/[trunk + pelvis], knee²/[trunk + pelvis], and trunk/pelvis), interaction terms (trunk × knee, trunk × pelvis, and pelvis × knee), and nonlinear terms (trunk², pelvis², and knee²). In addition, a composite feature (summated angle; trunk + pelvis + knee) was calculated. In total, 17 features (3 primary joint angles and 14 engineered variables) were used for subsequent model training. These ratios, interaction, nonlinear, and composite features were designed to capture coordination-related movement patterns beyond isolated joint deviations, potentially improving discrimination across knee-function levels and supporting clinical interpretability.

### Feature Selection

The dataset was divided into training and test sets using stratified sampling, with 70% (n=73) allocated for training and 30% (n=32) for independent testing, ensuring proportional representation of the 3 movement quality classes (good, moderate, and poor). All models were trained and evaluated using the original class distribution without oversampling or synthetic data generation techniques.

Feature selection was performed using a 2-stage hybrid approach to identify features that exhibited significant between-class differences while preserving biomechanically meaningful feature diversity. First, a filter-based method using a 1-way ANOVA *F_df_*-test with Benjamini-Hochberg false discovery rate correction was applied, resulting in the selection of 16 features that showed significant differences across the 3 movement quality classes (*P*<.05), with the trunk-to-pelvis ratio excluded because it did not show statistically significant between-class differences. Next, a wrapper-based method using recursive feature elimination with cross-validation and a random forest classifier was applied to the filtered feature set. Recursive feature elimination with cross-validation iteratively removed features that did not improve cross-validated classification performance, helping to mitigate functional redundancy among correlated variables derived from the same primary joint angles. Five-fold cross-validation was used with classification accuracy as the optimization metric. The final feature set was obtained by intersecting the results of both methods, resulting in 8 complementary features with reduced redundancy, including the knee valgus angle, ratio-based features (knee/trunk, knee/[trunk + pelvis], and knee²/[trunk + pelvis]), interaction terms (trunk × knee and pelvis × knee), nonlinear knee-related features (knee²), and a composite summated angle (trunk + pelvis + knee).

### Classifiers

All implementations were conducted in Python 3.9 (Python Software Foundation) using standard machine learning libraries. For models that are sensitive to feature scaling, such as logistic regression, SVM, and *k*-nearest neighbors (KNN), *z*-score normalization was applied within a pipeline, while tree-based models were trained on raw feature values. Seven classifiers, including logistic regression, SVM, KNN, random forest, gradient boosting, light gradient boosting machine (LightGBM), and adaptive boosting (AdaBoost), were tuned via grid search with stratified 5-fold cross-validation using the macro-averaged *F*_1_-score as the optimization metric. Hyperparameter search ranges included the regularization type and strength for logistic regression, the kernel type and penalty parameters for SVM, the number of neighbors and distance metrics for KNN, the tree depth and number of estimators for ensemble tree methods, and the learning rate for boosting algorithms.

### Model Evaluation

Model performance was evaluated using stratified 5-fold cross-validation on the training set and an independent held-out test set. Three primary metrics were used to assess classification performance: accuracy, macro-averaged *F*1-score, and multiclass area under the receiver operating characteristic curve (AUC). The macro-averaged *F*_1_-score was computed as the unweighted mean of the class-specific *F*_1_-scores, ensuring balanced evaluation across the 3 movement quality categories. Multiclass AUC was calculated using the one-vs-rest strategy, averaging the AUC scores obtained for each class.

### Model Explainability

Both SHAP and LIME analyses were conducted exclusively on the final trained model, ensuring consistency between the interpretability results and the model used for evaluation. This dual approach enabled the identification of class-specific feature importance at the global level and the extraction of instance-specific decision patterns at the local level, supporting potential clinical and fitness applications [24,25]. Global interpretability was assessed using SHAP, applied to the final machine learning model. SHAP values were computed for each of the 8 selected features to quantify feature contributions to the model’s class predictions. Class-wise SHAP value aggregation was used to identify the most influential features for each movement quality category.

Local interpretability was examined using LIME to generate instance-level explanations. LIME was applied to representative samples from each class in the test set, with the number of perturbed samples set to 5000 to ensure stability of the local surrogate models. The output from LIME was used to identify feature value ranges for each of the selected features that were most predictive of class membership. These thresholds were derived from the feature values at the decision boundaries learned by the model, providing interpretable biomechanical markers for distinguishing between “good,” “moderate,” and “poor” performance.

### Computational Feasibility

Model training was performed offline as a 1-time procedure using a standard central processing unit-based environment (Google Colab) without graphics processing unit acceleration. Due to the use of lightweight classifiers and a limited set of interpretable features, training typically completed within a few seconds. Inference was conducted at the participant level, with each video processed independently. The inference pipeline, which included pose estimation, feature extraction, and classification, typically required only a few seconds per video on a central processing unit. In the proposed framework, the smartphone was used primarily for video acquisition, while pose estimation, feature computation, and classification were performed on a personal computer. These findings indicated that the proposed pipeline has modest computational requirements and is feasible for practical implementation.

### Statistical Analysis

Descriptive statistics were computed for all participant characteristics and kinematic variables. Continuous data were reported as mean (SD), and categorical data were summarized as counts. Normality was assessed using the Shapiro-Wilk test. Differences among the 3 functional groups (good, moderate, and poor) were examined using 1-way ANOVA for continuous measures (age, BMI, weekly exercise duration, and peak frontal-plane angles). When a significant group effect was detected, Tukey honestly significant difference post hoc tests were conducted for pairwise comparisons. Statistical significance was defined as an *α* level of .05.

### Ethical Considerations

The study protocol was reviewed and approved by the Institutional Review Board of Jeonju University (jjIRB-210315-HR-2021-03210) in accordance with the Declaration of Helsinki and relevant ethical guidelines. All participants were fully informed about the study’s purpose, procedures, potential benefits, and rights prior to participation. Written informed consent was obtained from all participants prior to enrollment. All smartphone video data were collected solely for research purposes and were securely stored on an encrypted, password-protected server accessible only to the research team. To ensure privacy and confidentiality, all video and related data were deidentified; facial information and other personally identifiable details were neither analyzed nor disclosed. Participants were not asked to provide their real names; instead, each participant was assigned a unique anonymized code. Each participant received a small monetary incentive (approximately US $10) for participating in the study.

## Results

### Participant Characteristics

Participant characteristics are summarized in Table 1. The study included participants across the 3 SLS performance classification grades (good, moderate, and poor). While age, BMI, and weekly exercise duration did not significantly differ across groups, peak frontal-plane kinematics, including trunk lateral tilt, pelvic lateral tilt, and knee valgus, showed significant between-group differences (*P*<.05). Post hoc Tukey tests showed that trunk lateral tilt was significantly greater in the poor group than in the good and moderate groups, while no significant difference was observed between the good and moderate groups. Pelvic lateral tilt differed significantly between the good group and the other groups, whereas knee valgus increased stepwise across all 3 grades (*P*<.05).

**Table 1.** Participants’ characteristics (n=105).

	SLS^a^ performance grading criteria			
Characteristics	Good (0)	Moderate (1)	Poor (2)	*P* value
Number of participants, n	28	41	36	—^b^
Gender (men/women), n	13/15	21/20	19/17	—
Dominant side (right/left), n	26/2	40/1	35/1	—
Age (y)	22.36 (2.03)^c^	22.46 (2.85)	22.20 (2.47)	.75
BMI (kg/m^2^)	22.38 (3.35)	22.80 (3.59)	23.22 (3.33)	.53
Exercise duration per week (min)	445.42 (286.15)	353.25 (339.16)	331.57 (215.35)	.38
Trunk lateral tilt (°)^d^ ^e^ ^f^	6.00 (2.47)	7.24 (3.23)	10.24 (6.34)	<.001
Pelvic lateral tilt (°)^d^ ^f^ ^g^	6.31 (1.93)	8.50 (2.76)	10.38 (4.94)	<.001
Knee valgus (°)^d^ ^e^ ^f^ ^g^	−2.63 (2.55)^h^	3.67 (3.75)	13.24 (9.71)	<.001

a SLS: single-leg squat.

b Not available.

c Mean (SD).

d Peak joint angles measured during the single-leg squat test.

e Post hoc showed a significant difference between moderate and poor groups (*P*<.05).

f Post hoc showed a significant difference between good and poor groups (*P*<.05).

g Post hoc showed a significant difference between good and moderate groups (*P*<.05).

h Negative values represent lateral deviation (knee varus) from neutral alignment, whereas positive values represent medial deviation (knee valgus).

### Classification Performance of Machine Learning Models

**Figure 4.**
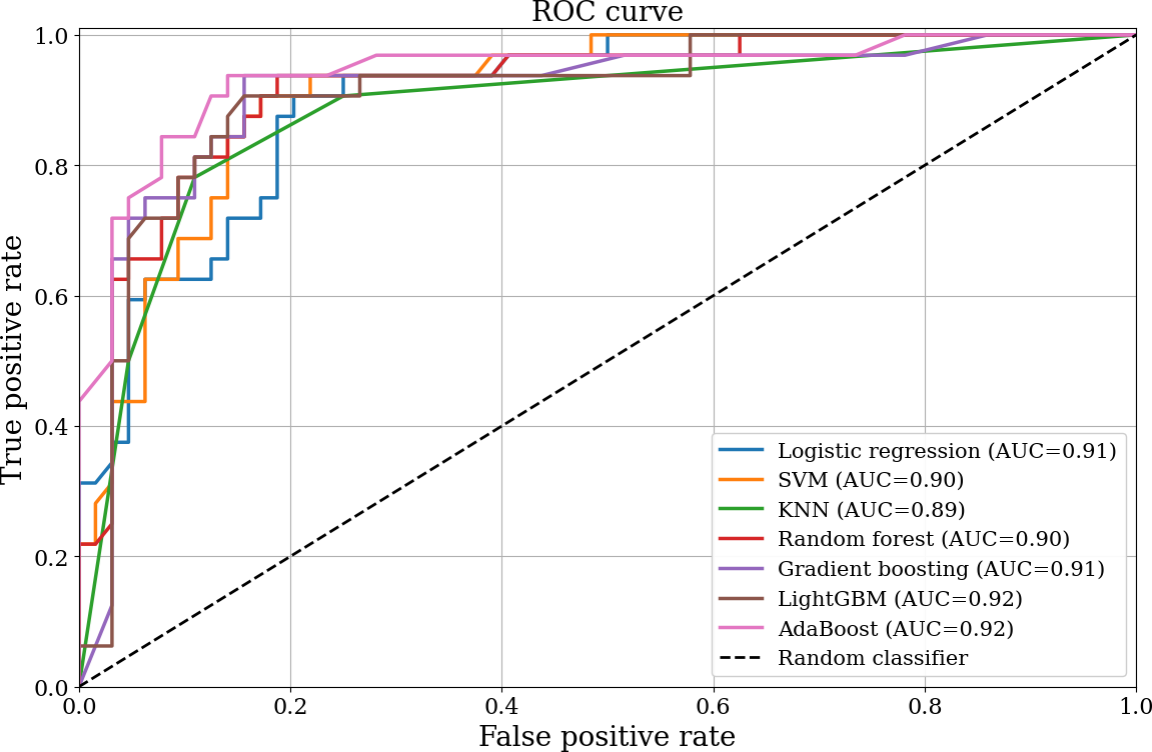
ROC curves and corresponding AUC values. AdaBoost: adaptive boosting; AUC: area under the curve; KNN: *k*-nearest neighbors; LightGBM: light gradient boosting machine; ROC: receiver operating characteristic; SVM: support vector machine.

Seven individual classifiers were evaluated using stratified 5-fold cross-validation, and final performance was assessed on a held-out test set (Table 2). AdaBoost demonstrated the best overall performance (AUC=0.92), comparable to LightGBM (AUC=0.92) and slightly higher than gradient boosting (AUC=0.91) (Figure 4). Accordingly, AdaBoost was selected as the final classifier for subsequent analysis. To confirm the value of feature selection, we compared AdaBoost performance using the full 17-feature pool versus the final 8 selected features. The AUC remained comparable (0.92 vs 0.92), indicating that the compact feature set retained most of the predictive information while improving model simplicity and interpretability.

**Figure 5.**
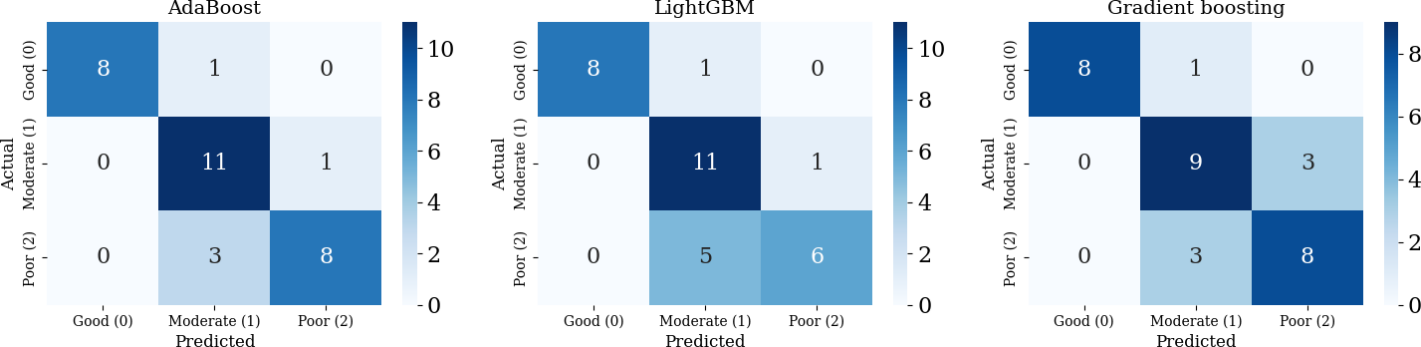
Confusion matrices of AdaBoost, LightGBM, and gradient boosting models. AdaBoost: adaptive boosting; LightGBM: light gradient boosting machine.

The confusion matrices for AdaBoost, LightGBM, and gradient boosting are presented in Figure 5. Overall, AdaBoost showed more consistent class-wise discrimination, with reduced confusion between adjacent functional grades (particularly moderate and poor) compared with the other models, consistent with its overall performance metrics.

**Table 2.** Performance metrics of the evaluated classifiers.

Classifier	Accuracy	*F*_1_-score	AUC^a^	Sensitivity	Specificity
Logistic regression	0.69	0.70	0.91	0.69	0.83
Support vector machine	0.75	0.76	0.90	0.75	0.83
k‑nearest neighbors	0.78	0.79	0.89	0.79	0.88
Random forest	0.75	0.76	0.90	0.75	0.87
Gradient boosting	0.78	0.80	0.91	0.79	0.89
LightGBM^b^	0.81	0.82	0.92	0.81	0.90
AdaBoost^c^	0.84	0.85	0.92	0.84	0.92

a AUC: area under curve.

b LightGBM: light gradient boosting machine.

c AdaBoost: adaptive boosting.

### Key Feature Analysis and Model Interpretability

**Figure 6.**
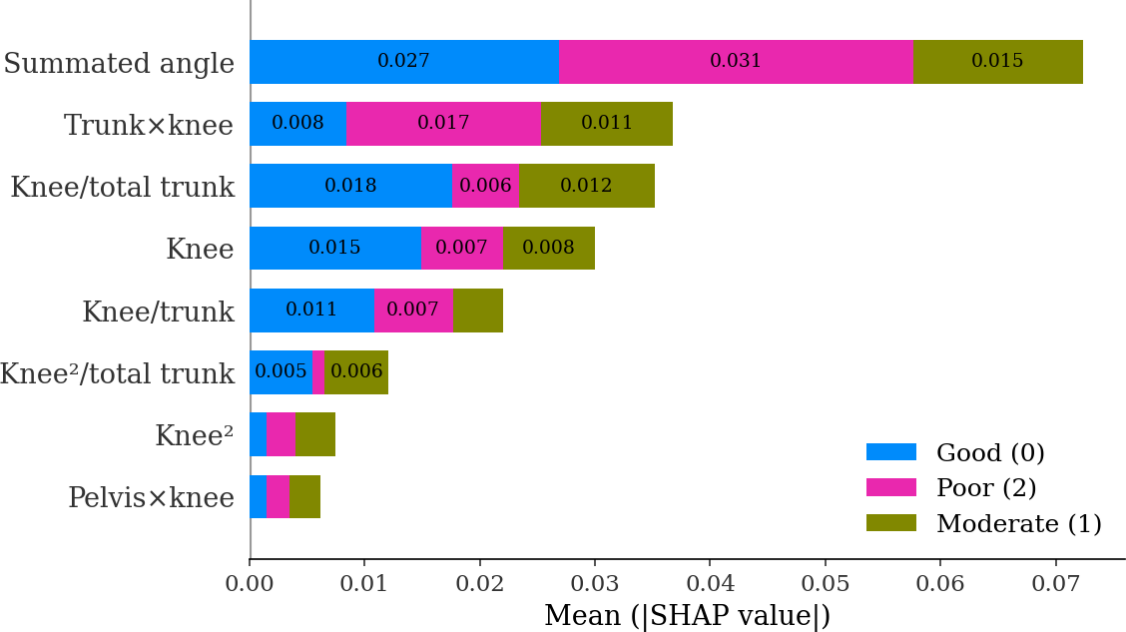
SHAP summary plot showing global feature importance for the final classification model, with class labels representing single-leg squat performance levels (class 0=good, class 1=moderate, class 2=poor; total trunk=trunk + pelvis). SHAP: Shapley additive explanations.

**Figure 7.**
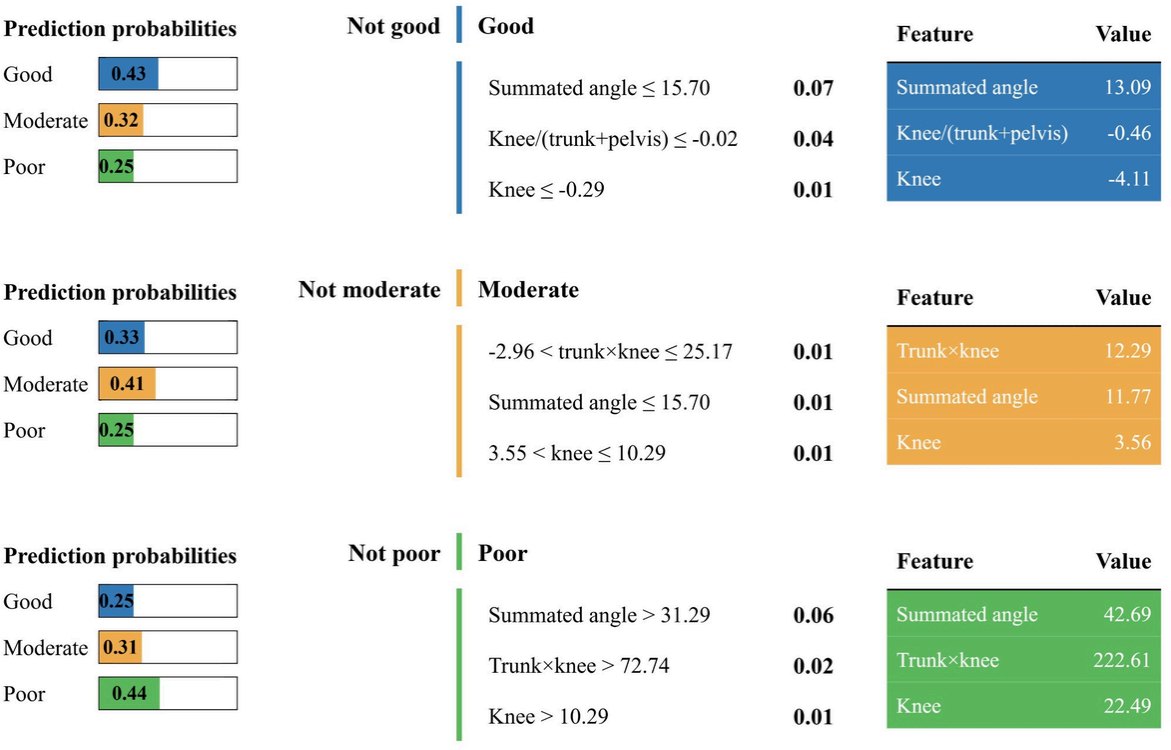
Local interpretable model-agnostic explanations-based instance-level explanations of feature contributions for representative samples from each class (good, moderate, and poor) classified by the final model.

To interpret the final classification model, we conducted both global and local interpretability analyses. SHAP identified the summated angle as the most influential feature, followed by the trunk × knee interaction, the knee-to-trunk ratio, and the knee angle (Figure 6). These results suggested that model predictions were influenced by both isolated knee deviations and intersegment coordination patterns, rather than conventional guideline-based single-joint criteria alone. For local interpretability, LIME provided case-specific explanations by visualizing how these features contributed to individual classifications across the 3 functional grades (Figure 7).

## Discussion

### Principal Findings

This study developed and evaluated an interpretable machine learning framework that classifies SLS performance into 3 levels (good, moderate, and poor) from single-smartphone, frontal-view SLS videos. Using a small set of biomechanically meaningful engineered features (summated angle, knee × trunk interaction, knee-to-trunk ratio, and knee angle), the AdaBoost classifier showed the best overall performance on a held-out test set (accuracy=0.84, *F*_1_-score=0.85, and AUC=0.92). SHAP and LIME enabled feature-based interpretation, allowing classification results to be translated into clinically interpretable feedback rather than labels alone. Importantly, the primary contribution of this study lies not in proposing a new algorithm but in demonstrating through a machine learning approach that impaired SLS performance is associated with coordination-related movement patterns rather than isolated joint deviations. By emphasizing the relative and interactive organization of trunk and knee movements, this coordination-based interpretation complements conventional guideline-driven SLS assessment and provides practical insights for rehabilitation decision-making and athletic screening.

Previous studies have reported that both wearable sensor-based and camera-based markerless models achieved moderate accuracy, typically ranging from 66% to 99% with an AUC of approximately 0.73, and often showed limited generalizability in 3-class SLS classification [9-11,26]. Notably, a recent study reported classification accuracies close to 99% using a single thigh-mounted IMU and a convolutional neural network-gated recurrent unit model trained on experimentally induced squat error patterns [26]. However, this IMU-based convolutional neural network-gated recurrent unit approach operates as a largely black-box model and relies on experimentally induced error motions (knee valgus/varus or excessive trunk flexion/extension), which may limit interpretability and ecological validity for clinical decision-making and real-world functional screening. In contrast, the present study emphasizes clinical translation by training the model on naturally performed SLS trials, with expert clinicians selecting representative videos that reflect real-world movement quality rather than artificially induced deviations. To move beyond guideline-based or composite measures, we generated ratio-, interaction-, and nonlinear-based features to capture interjoint coordination and identified summated angle, knee × trunk, knee-to-trunk ratio, and knee angle as the most informative predictors, thereby broadening the biomechanical basis for SLS assessment. By combining expert-labeled functional outcomes with interpretable feature representations learned from natural, nonexperimentally induced SLS performances, the proposed framework was designed to support clinical reasoning with transparent decision logic. Furthermore, machine learning models must be trained on data that are realistically obtainable in routine practice; otherwise, even high-performing algorithms remain difficult to translate when they depend on non-consumer sensors, multiple cameras, or specialized laboratory setups [27]. The present framework addresses this limitation by relying exclusively on a single, frontal smartphone video to extract interpretable kinematic features during standard SLS testing without specialized IMU sensor. This design enables automatic classification of SLS performance levels while maintaining practical feasibility, interpretability, and direct applicability in real-world sports and rehabilitation settings. Therefore, the key novelty lies not only in performance metrics but also in an ecologically valid and explainable approach that can be realistically implemented using smartphone video in routine screening workflows.

Beyond predictive accuracy, the explainability analyses provided additional biomechanical insights into SLS performance. SHAP highlighted the summated angle as the most influential feature, with additional contributions from knee-specific and coordination-related metrics, including the knee × trunk interaction, the knee-to-trunk ratio, and knee angle. These findings suggest that impaired performance is associated with multisegment movement patterns, rather than isolated joint deviation alone. Previous studies using individual joint or composite measures achieved only moderate accuracy (66% with an SVM and 76.9% with a decision tree) [9,10], without considering intersegmental interactions. In contrast, our approach explicitly incorporated coordination-related engineered features and combined them with an interpretable machine learning model, enabling biomechanically grounded explanations beyond conventional joint-by-joint criteria. This observation aligns with biomechanical evidence that interjoint coordination patterns are critical markers of movement quality during squatting and single-leg tasks [7], and supports the role of trunk-knee coupling as a central determinant of functional impairment [28]. Complementary LIME analyses further illustrated instance-specific decision boundaries, showing that case-level classifications were consistently explained by combinations of summated angle, coordination-related metrics, and knee angle supporting transparent subject-level interpretation. By clarifying why a patient is classified as having poor SLS performance, the XAI results may help clinicians and trainers gain confidence in machine learning-based SLS evaluation. Moreover, they can guide expert decisions on whether management strategies should focus solely on the knee or also address altered trunk-knee coordination, thereby supporting more personalized rehabilitation and remote monitoring.

### Practical Implications

The practical meaning of this study is that it enables a low-cost, scalable, and explainable workflow for screening SLS performance using smartphone-recorded SLS videos, providing interpretable decision support rather than simple movement monitoring. In team sports, athletic trainers can apply this workflow for periodic screening, reducing the burden of manually reviewing large volumes of videos, grading SLS performance, and producing narrative reports for multiple athletes. Importantly, SHAP- and LIME-based explanations support interpretation beyond the final grade by clarifying whether impaired performance is driven primarily by knee deviation, altered trunk control, or their coordination-related interaction, thereby informing targeted preventive or corrective strategies. In physical therapy settings, clinicians can similarly use the framework during intake or follow-up visits to obtain an objective functional grade with interpretable feature-based rationale, supporting individualized rehabilitation planning and progress tracking. Compared with rule-based fitness screening applications, the proposed machine learning approach better captures coordination-related movement patterns and provides consistent decision logic with interpretable explanations, supporting standardized documentation and scalable musculoskeletal screening in sports and rehabilitation settings.

### Limitations

Despite these promising results, several limitations should be acknowledged. First, the dataset size was relatively small (n=105 participants). However, prediction instability analyses suggested that model performance stabilized with the available sample [15], and stratified cross-validation and systematic feature selection were applied to assess the stability of the results [13]. Nevertheless, future studies should validate the model in larger cohorts with external testing to quantify generalizability across populations and real-world settings. Second, all videos were recorded using the same smartphone under a standardized frontal-view setup with fixed camera distance and height, consistent with routine clinician-guided SLS screening protocols. While this standardized design improves reliability and reduces measurement variability, it may limit generalizability to home- or telehealth-based recordings where camera setup and recording conditions are less controlled. In particular, variations in smartphone camera specifications and video quality (resolution and frame rate), as well as real-world environmental factors such as lighting and background clutter, may affect pose estimation accuracy and introduce noise into extracted kinematic features. Future work should quantify sensitivity by systematically varying recording conditions (smartphone model, lighting, background, and camera distance or height) and examining the impact on pose estimation stability and final classification performance. Third, participants were limited to young healthy adults, which may restrict generalizability to clinical populations and athletic cohorts. Future studies should evaluate performance in more diverse groups, including older adults, patients with knee-related conditions, and injured athletes, to support clinical translation. Finally, because the dataset showed a right-skewed dominant-side distribution (Table 1), future studies should include more left-dominant participants and evaluate left-right normalization or symmetry-based augmentation strategies to improve model robustness.

### Conclusion

This study developed an interpretable machine learning framework for classifying SLS performance into 3 levels (good, moderate, and poor) from frontal-view SLS videos recorded with a single smartphone. Using a small set of biomechanically meaningful engineered features (summated angle, knee × trunk interaction, knee-to-trunk ratio, and knee angle), the AdaBoost classifier achieved good overall performance on a held-out test set. SHAP and LIME provided consistent global and local explanations, enabling transparent interpretation of SLS performance classifications. Importantly, the proposed coordination-informed feature design provides a distinct contribution by reframing SLS assessment beyond isolated joint deviations, instead emphasizing intersegment movement patterns that align more closely with clinical reasoning and athletic screening. This workflow uses smartphones only for standardized video acquisition, while explainable SLS performance screening is achieved through an interpretable machine learning framework. Given the lightweight feature set and classical machine learning models used, the proposed framework is suitable for future on-device inference on modern smartphones, which may support rehabilitation planning and injury prevention strategies.

## Abbreviations

AdaBoost: adaptive boosting
AUC: area under curve
IMU: inertial measurement unit
LightGBM: light gradient boosting machine
LIME: local interpretable model-agnostic explanations
SHAP: Shapley additive explanations
SLS: single-leg squat
SVM: support vector machine
XAI: explainable artificial intelligence
